# The Effects and Mechanism of Atorvastatin on Pulmonary Hypertension Due to Left Heart Disease

**DOI:** 10.1371/journal.pone.0157171

**Published:** 2016-07-07

**Authors:** Qing Wang, Yi-Zhan Guo, Yi-Tao Zhang, Jiao-Jie Xue, Zhi-Chong Chen, Shi-Yao Cheng, Mao-De Ou, Kang-Lin Cheng, Wei-Jie Zeng

**Affiliations:** 1 Department of Cardiology, The Sixth Affiliated Hospital of Sun Yat-sen University, Guangzhou, Guangdong, China; 2 Department of Hepatic Surgery, The First Affiliated Hospital of Sun Yat-sen University, Guangzhou, Guangdong, China; Augusta University, UNITED STATES

## Abstract

**Background:**

Pulmonary hypertension due to left heart disease (PH-LHD) is one of the most common forms of PH, termed group 2 PH. Atorvastatin exerts beneficial effects on the structural remodeling of the lung in ischemic heart failure. However, few studies have investigated the effects of atorvastatin on PH due to left heart failure induced by overload.

**Methods:**

Group 2 PH was induced in animals by aortic banding. Rats (n = 20) were randomly divided into four groups: a control group (C), an aortic banding group (AOB_63_), an atorvastatin prevention group (AOB_63_/ATOR_63_) and an atorvastatin reversal group (AOB_63_/ATOR_50-63_). Atorvastatin was administered for 63 days after banding to the rats in the AOB_63_/ATOR_63_ group and from days 50 to 63 to the rats in the AOB_63_/ATOR_50-63_ group.

**Results:**

Compared with the controls, significant increases in the mean pulmonary arterial pressure, pulmonary arteriolar medial thickening, biventricular cardiac hypertrophy, wet and dry weights of the right middle lung, percentage of PCNA-positive vascular smooth muscle cells, inflammatory infiltration and expression of RhoA and Rho-kinase II were observed in the AOB_63_ group, and these changes concomitant with significant decreases in the percentage of TUNEL-positive vascular smooth muscle cells. Treatment of the rats in the AOB_63_/ATOR_63_ group with atorvastatin at a dose of 10 mg/kg/day significantly decreased the mean pulmonary arterial pressure, right ventricular hypertrophy, pulmonary arteriolar medial thickness, inflammatory infiltration, percentage of PCNA-positive cells and pulmonary expression of RhoA and Rho-kinase II and significantly augmented the percentage of TUNEL-positive cells compared with the AOB_63_ group. However, only a trend of improvement in pulmonary vascular remodeling was detected in the AOB_63_/ATOR_50-63_ group.

**Conclusions:**

Atorvastatin prevents pulmonary vascular remodeling in the PH-LHD model by down-regulating the expression of RhoA/Rho kinase, by inhibiting the proliferation and increasing the apoptosis of pulmonary arterial smooth muscle cells, and by attenuating the inflammation of pulmonary arteries.

## Introduction

Pulmonary hypertension due to left heart disease (PH-LHD) is one of the most common forms of PH. Both a “passive” increase in pulmonary vascular pressures and a concomitant increase in pulmonary vascular resistance contribute to the pathogenesis of PH-LHD. The mechanisms responsible for the increased pulmonary vascular resistance involve pulmonary vascular remodeling. Pulmonary arterial hypertension (PAH)-targeted therapies, including prostanoids, endothelin receptor antagonists and phosphodiesterase type 5 inhibitors, have been approved for the treatment of PAH in recent decades. However, approved targeted therapies for PH-LHD are lacking. Limited clinical data support the hypothesis that sildenafil improves the functional capacity and clinical status of PH-LHD patients [1–4]. However, the use of sildenafil in PH-LHD has not been demonstrated to be effective in large randomized clinical trials. Furthermore, prostacyclin therapy and endothelin receptor antagonists have been shown to be harmful in clinical trials [5,6]. Therefore, there is a need for effective drug treatments.

RhoA/Rho-kinase is reportedly involved in the pathogenesis of various cardiovascular diseases, including PH-LHD [7–9]. RhoA is a small GTPase protein, and Rho-kinase is one of its main downstream effectors. The binding of RhoA to the Rho-binding domain of Rho-kinase activates regulatory cell functions, including the proliferation, migration and contraction of vascular smooth muscle cells (VSMCs). Therefore, the inhibition of Rho-kinase may prevent and attenuate the development of PH-LHD [9], and the administration of the Rho-kinase inhibitor fasudil was recently reported to attenuate pulmonary hypertension, right ventricular hypertrophy and pulmonary arteriolar medial thickness [9].

Statins are inhibitors of the key enzyme of cholesterol synthesis, 3-hydroxy-3-methylglutaryl coenzyme A (HMG-CoA) reductase, and exert antiproliferative effects. HMG-CoA reductase is essential for the synthesis of isoprenoids, which are required for the post-translational isoprenylation of Rho and Ras family GTPases. Previous studies have demonstrated that statins improve PH induced by monocrotaline or hypoxia through the RhoA/Rho-kinase pathway [10]. Recently, atorvastatin was reported to exert beneficial effects on the structural remodeling of the lung in ischemic heart failure [11]. However, few studies have been performed on PH due to left heart failure induced by overload.

In the present study, we evaluated the potential role of atorvastatin on pulmonary vascular remodeling and right ventricular hypertrophy in rats with left heart disease induced by transverse aortic constriction and investigated the potential mechanisms of action of atorvastatin.

## Materials and Methods

### Animal model of PH

All animal protocols were approved by the Animal Ethics and Research Committee of the Sun Yat-sen University (22014016). PH was induced in male Sprague-Dawley rats (with a body weight of 250–300 g) that were randomly assigned to aortic-banded groups. All of the rats were fed in a specific pathogen-free area with a set temperature of (22±2)°C and a moisture level of (55±5)%. The rats were fed standard solid food and sterilized water, and light was supplied 12 h per day. A left thoracotomy in the third intercostal space was performed under respiratory support with a small animal ventilator (RWD, Shenzhen, China). After the animals were anesthetized with pentobarbital (36 mg/kg, i.p.), the skin and muscle were separated. A blunt syringe needle (inner diameter of 1.2 mm) was placed along the axis of the ascending aorta, and a length of 1–0 nylon suture was tied approximately 1 cm distal to the aortic valve around the aorta. The needle was then removed, leaving a stenosis that could be directly observed. The sham-operated rats underwent the same operation with the exception of aorta banding.

The rats (n = 20) were randomly divided into four groups: a control group (C), an aorta banding group (AOB_63_), an atorvastatin prevention group (AOB_63_/ATOR_63_) and an atorvastatin reversal group (AOB_63_/ATOR_50-63_). The rats in both the C group (n = 5) and the AOB_63_ group (n = 5) were administered saline (0.5 ml/100 g/day) for 63 days. The rats in the AOB_63_/ATOR_63_ group (n = 5) were administered atorvastatin (10 mg/kg/day, 0.5 ml/100 g/day) for 63 days after banding. The rats in the atorvastatin reversal group (AOB_63_/ATOR_50-63_; n = 5) were administered saline from Day 1 to Day 49 and atorvastatin from Day 50 to Day 63. On Day 64, the mean pulmonary pressures of all of the rats were measured, and the rats were then sacrificed.

### Hemodynamic measurements

After the animals were anesthetized with pentobarbital (36 mg/kg, i.p), a pulmonary artery catheter (OD = 0.85 mm, ID = 0.42 mm) filled with 1 ml of heparin solution (1000 IU/ml) was inserted into the right external jugular vein, the superior vena cava, the right atrium, the right ventricle and the pulmonary artery in turn. The catheter was connected to a pressure transducer, and pressure data were recorded with a polygraph system BL-420E (Taimeng, Chengdu, China).

### Tissue preparation

After the rats were sacrificed with pentobarbital, the middle lobe of the right lung was removed to measure the wet-to-dry-weight ratio. After removing the right middle lobe, the lung and heart were perfused with saline and removed. The other lobes of the right lung were fixed in 4% paraformaldehyde for 24 h, embedded in paraffin and sectioned. Hematoxylin and eosin (H-E) staining was subsequently performed, and α-SMA, PCNA, Mac-2 and LY-6G were examined by immunohistochemistry. A TUNEL assay was also performed to measure the degree of SMC apoptosis. The left lung was frozen in liquid nitrogen and stored at -80°C for western blot analysis. The right ventricle (RV) and the left ventricle (LV) with the interventricular septum (IVS) were isolated and weighed. The ratios of RV to body weight (BW) and LV+IVS to BW were measured to determine the extent of ventricle hypertrophy.

### Western blotting for RhoA and ROCK II

The lung tissues were lysed on ice with RIPA buffer (1% Triton X-100, pH 7.4, 150 mM NaCl, 1% sodium deoxycholate, 0.1% SDS, 1 mM sodium orthovanadate, and 10 mM EDTA) supplemented with protease inhibitors (KeyGen, Nanjing, China) and phosphatase inhibitors (KeyGen, Nanjing, China). After the protein concentrations were measured through the BCA method, equal amounts of protein were electrophoresed on a 12% sodium dodecyl sulfate-polyacrylamide gel and transferred onto a PVDF membrane (Millipore, Billerica, MA, USA). The membrane was blocked with 5% BSA Tris-buffered saline (pH 7.6, containing 0.1% Tween and 5% bovine serum albumin) for 1 h at room temperature and probed with anti-RhoA antibodies (1:300, Santa Cruz Biotechnology, Santa Cruz, CA, USA) and then with the secondary antibody for 1 h at room temperature. The protein of interest on the membrane was detected using an enhanced chemiluminescence (ECL) detection system (KeyGen, Nanjing, China).

To examine the expression of Rho-kinase II (ROCK II), the same protocols with the following exceptions were followed: an 8% sodium dodecyl sulfate-polyacrylamide gel was used, and the membrane was probed with anti-ROCK II (1:300, Santa Cruz, CA, USA) antibodies.

### Pulmonary arteriole remodeling, proliferation and apoptosis

After hematoxylin and eosin (HE) staining, 10 areas of pulmonary arterioles approximately 50 μm to 100 μm in diameter were randomly chosen, and the medial wall thickness was measured under a microscope at 400× magnification using the following equation: percent wall thickness (WT%) = (medial thickness×2/external diameter) ×100%.

The relative level of pulmonary vascular muscularization was determined by immunohistochemical staining with an anti-α-SMA antibody. Briefly, tissue sections (4 μm) were deparaffinized, rehydrated, subjected to antigen retrieval in Tris-EDTA buffer (pH 8.0) for 23 min at 95–100°C and washed in PBS (pH 7.4). The sections were incubated with 3% hydrogen peroxide in PBS for 20 min and then with a 3% BSA solution for 30 min. The sections were incubated with a mouse monoclonal anti-α-SMA antibody (1:100, Guge, Wuhan, China) overnight at 4°C and then with an avidin/biotin peroxidase-linked secondary antibody (DAKO, Glostrup, Denmark). The staining was visualized using an EnVision Detection System (DAKO, Glostrup, Denmark). For each rat, 20 intra-acinar arteries less than 50 μm in diameter were examined and categorized as non-muscular (NM), partially muscular (PM) or fully muscular (FM). The muscularization scores (M scores) were calculated to differentiate the level of muscularization of the arteries. NM, PM and FM arteries were given scores of 0, 1 and 2.

The proliferation and apoptosis rates of SMCs were measured by immunohistochemistry staining with anti-PCNA antibody (1:100, Guge, Wuhan, China) and a terminal deoxynucleotidyl-transferase-mediated dUTP nick end-labeling (TUNEL) assay (Roche Diagnostics, Mannheim, Germany), respectively. These assays were performed according to the instructions provided by the manufacturers. The TUNEL-positive SMCs in at least 20 optical fields were counted for each specimen. The apoptotic rate is expressed as the percentage of apoptotic cells in the total population of SMCs per field.

### Inflammation of pulmonary arterioles

To evaluate the level of inflammation, inflammation scores (I scores) ranging from 0 to 4 were given based on the HE staining: 0 indicated no inflammation, 1 indicated ≤25% inflammation, 2 indicated >25% and ≤50% inflammation, 3 indicated >50% and ≤75% inflammation, and 4 indicated >75% inflammation. Immunohistochemistry for Mac-2 and LY-6G was performed to assess the distribution of macrophages and neutrophil granulocytes.

### Statistical analysis

The data are presented as the means±SD. Western blots were analyzed by densitometry. All of the data from the four groups were analyzed by ANOVA followed by Bonferroni. P<0.05 was considered statistically significant. All of the statistical tests were performed using SPSS v13.0 (SPSS Inc., Chicago, IL, USA).

## Results

### Effects of atorvastatin on the mean pulmonary arterial pressure, ventricular hypertrophy and W/D weight of the lung

**Fig 1:**
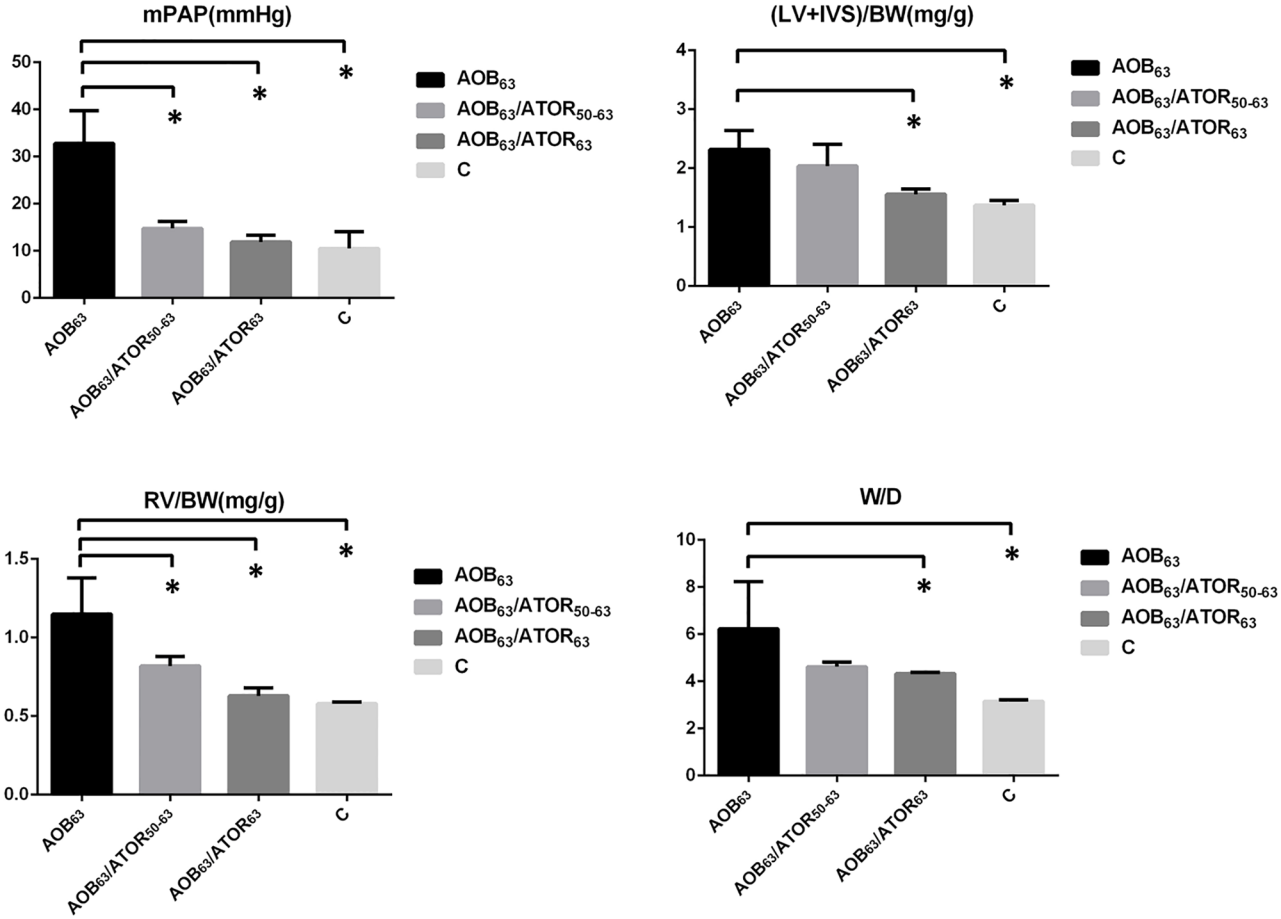
Comparison of the Hemodynamics, Ventricle Weights and Lung Weights among the Groups. *P<0.05 compared with the AOB_63_ group.

As presented in Table 1 and Fig 1, the mean pulmonary arterial pressure (mPAP) was significantly higher in the AOB_63_ group compared with the control group (32.82±6.96 vs. 10.53±3.52), indicating that the model of PH induced by aortic banding mimicked the human disease. The mPAP was significantly decreased in the AOB_63_/ATOR_63_ and AOB_63_/ATOR_50-63_ groups compared with the AOB_63_ group.

**Table 1:** Comparison of the Hemodynamics, Ventricle Weights and Lung Weights among the Groups.

	AOB_63_	AOB_63_/ATOR_50-63_	AOB_63_/ATOR_63_	C
mPAP (mmHg)	32.82±6.96	14.79±1.44*	11.88±1.43*	10.53±3.52*
LV+IVS/BW (mg/g)	2.32±0.32	2.04±0.37	1.56±0.09*	1.37±0.08*
RV/BW (mg/g)	1.15±0.23	0.82±0.06*	0.63±0.05*	0.58±0.01*
W/D	6.23±2.00	4.62±0.20	4.33±0.06*	3.15±0.07*

AOB_63_, aortic-banded rats not treated with atorvastatin; AOB_63_/ATOR_50-63_, aortic-banded rats administered atorvastatin from days 50 to 63; AOB_63_/ATOR_63_, aortic-banded rats administered atorvastatin for 63 days; C, control rats not subjected to aortic banding

*P<0.05 compared with the AOB_63_ group

Compared with the control group, the rats subjected to aortic banding exhibited significant increases in the RV/BW (1.15±0.23 vs. 0.58±0.01, *P*<0.05) and LV+IVS/BW ratios (2.32±0.32 vs. 1.37±0.08, *P*<0.05). The oral administration of atorvastatin for 63 days to the aortic-banded rats significantly decreased the RV/BW and LV+IVS/BW ratios (0.63±0.05 and 1.56±0.09, respectively, both *P*<0.05). However, only a trend toward a decrease in the LV+IVS/BW ratio was observed in the atorvastatin reversal group.

Compared with the sham-operated group, the W/D ratio (wet-to-dry-weight ratio of the right middle lung, respectively) was significantly higher in the AOB_63_ group, and this increase was attenuated in the AOB_63_/ATOR_63_ group (*P*<0.05).

### Effects of atorvastatin on pulmonary vascular remodeling, proliferation and apoptosis

**Fig 2:**
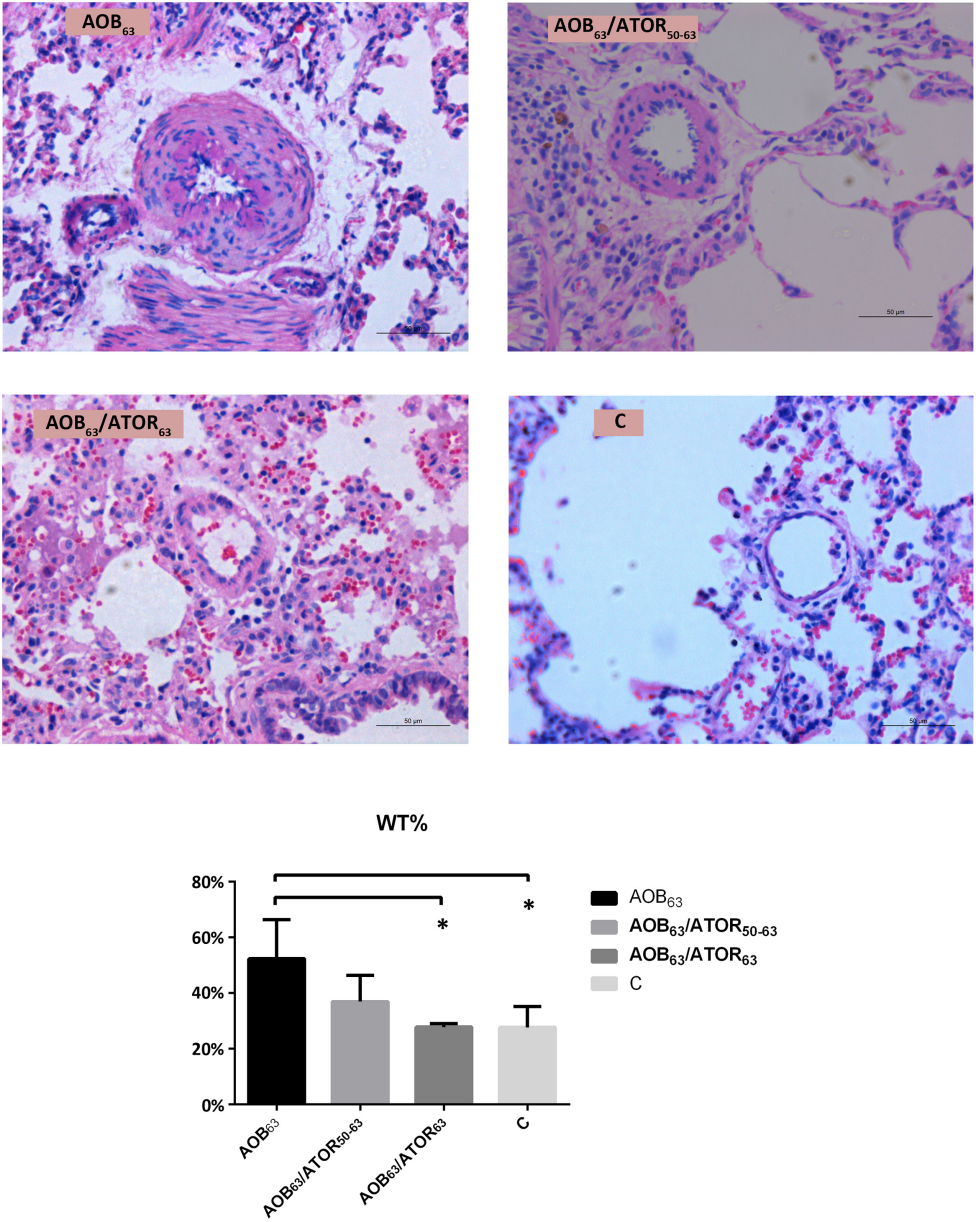
Comparison of the Percent Wall Thickness of the Pulmonary Arterioles among the Groups. HE staining of the lung tissue (magnification 400×, bar = 50 μm) demonstrated that the medial wall thickness of the pulmonary vasculature was increased in the AOB_63_ group compared with the C group. Treatment with atorvastatin attenuated the vascular remodeling in the AOB_63_/ATOR_63_ group. *P<0.05 compared with the AOB_63_ group.

As presented in Fig 2, the medial wall thickness of the pulmonary vasculature was increased in the AOB_63_ group compared with the control group (52.35±13.99% vs. 27.73±7.45%, *P*<0.05). Treatment with atorvastatin attenuated this vascular remodeling in the AOB_63_/ATOR_63_ group, which presented a decrease of approximately 24.53% compared with the AOB_63_ group (*P*<0.05). However, the medial wall thickness of the pulmonary vasculature in the AOB_63_/ATOR_50-63_ group was not decreased significantly compared with the AOB_63_ group.

**Fig 3:**
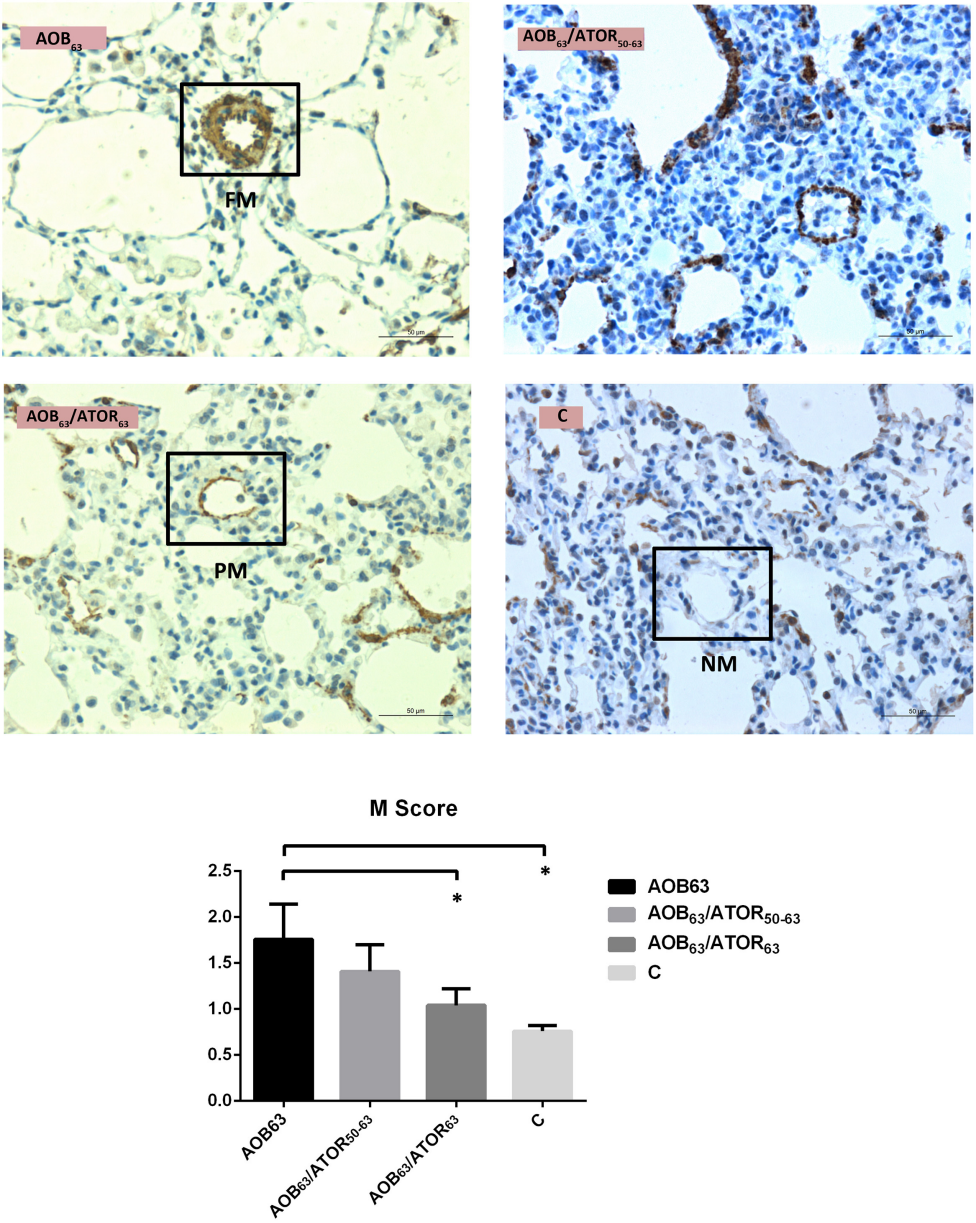
Comparison of the Muscularization Scores of the Pulmonary Arterioles among the Groups. Immunohistochemical staining of the lung tissue (magnification 400×, bar = 50 μm) revealed more abundant α-SMA in the AOB_63_ group compared with the C group, and the M score of the AOB_63_ group was higher. Treatment of the rats in the AOB_63_/ATOR_63_ group with atorvastatin attenuated the muscularization. (NM = non-muscular, PM = partially muscular, FM = fully muscular). *P<0.05 compared with the AOB_63_ group.

The percentage of muscularization of the pulmonary arterioles in the AOB_63_ group was significantly higher than that of the control group, as indicated by the M score (1.76±0.38 vs. 0.76±0.06, *P*<0.05). Compared with the AOB_63_ group, a significant reduction in the M score was detected in the AOB_63_/ATOR_63_ group (*P*<0.05, Fig 3).

**Fig 4:**
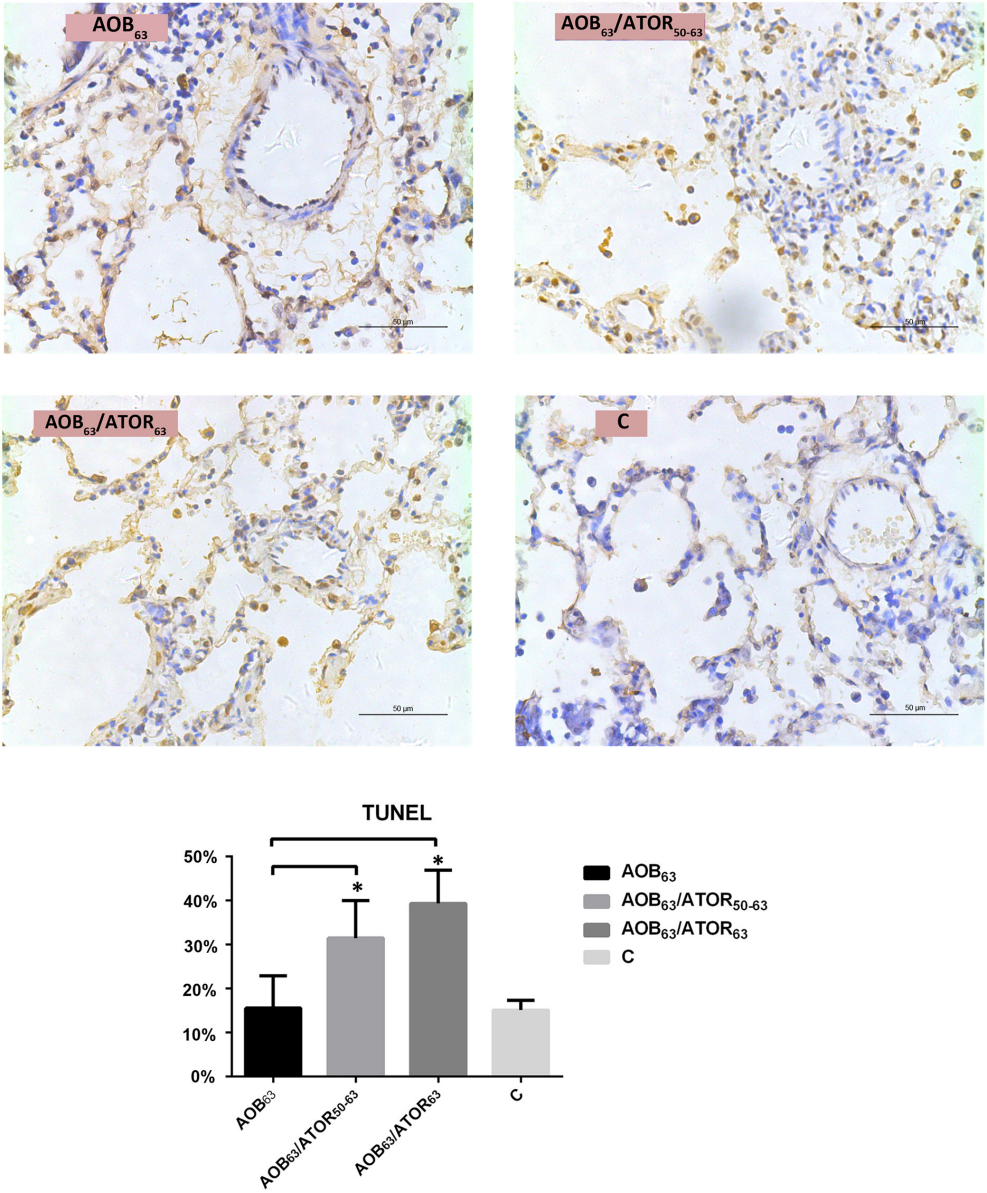
Comparison of TUNEL-Positive Cells and Percentage of Pulmonary Arterioles among the Groups. The percentage of TUNEL-positive cells (magnification 400×, bar = 50 μm) was significantly increased in both the AOB_63_/ATOR_63_ and AOB_63_/ATOR_50-63_ groups. *P<0.05 compared with the AOB_63_ group.

As shown in Fig 4, the mean percentage of TUNEL-positive VSMCs was 15.56% in the AOB_63_ group, and the percentage of TUNEL-positive cells was significantly increased in both the AOB_63_/ATOR_63_ and AOB_63_/ATOR_50-63_ groups (39.34% and 31.49%, respectively).

**Fig 5:**
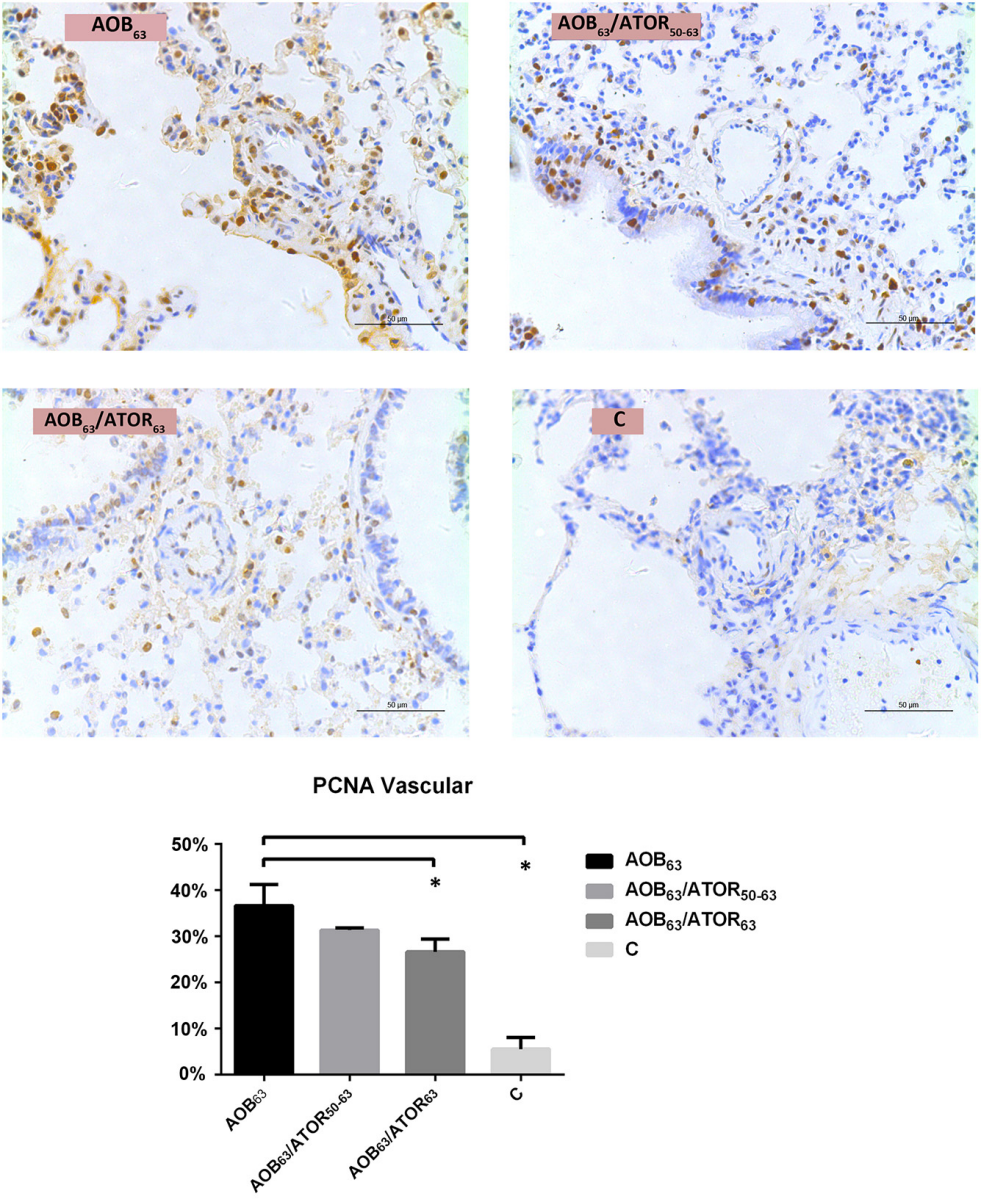
Comparison of PCNA-Positive Cells and Percentage of Pulmonary Arterioles among the Groups. The mean percentage of PCNA-positive cells (magnification 400×, bar = 50 μm) was significantly decreased in the AOB_63_/ATOR_63_ group. *P<0.05 compared with the AOB_63_ group.

The mean rate of proliferation (percentage of PCNA-positive cells) in the AOB_63_ group was 36.69%, and the mean percentage of PCNA-positive cells was significantly decreased in the AOB_63_/ATOR_63_ group (26.64%, *P*<0.05, Fig 5).

### Effects of atorvastatin on inflammation

**Fig 6:**
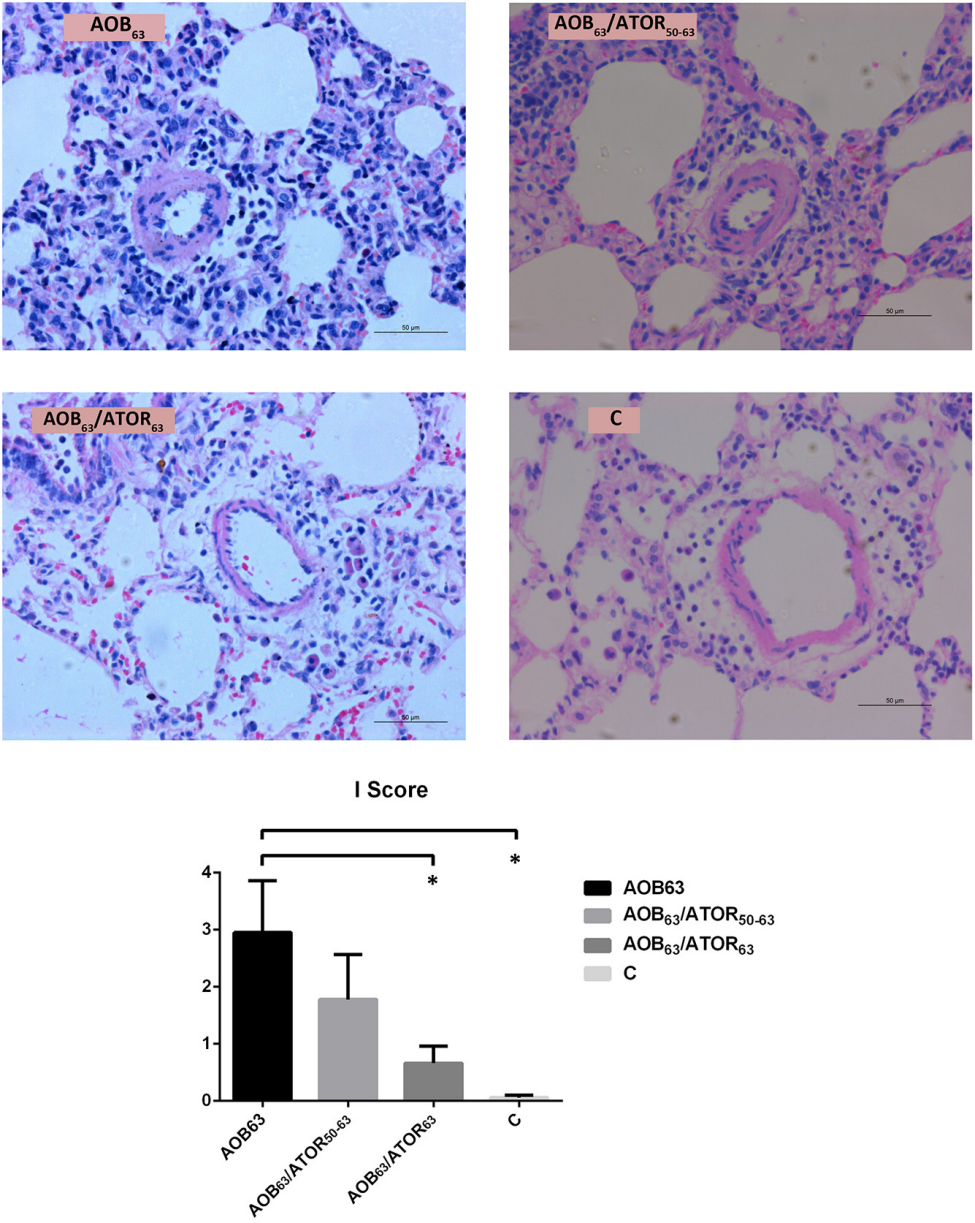
Comparison of the Inflammation Scores of Pulmonary Arterioles among the Groups. HE staining of the lung tissue (magnification 400×, bar = 50 μm) revealed that the I score was significantly attenuated in the AOB_63_/ATOR_63_ group relative to the AOB_63_ group. *P<0.05 compared with the AOB_63_ group.

The presence of inflammation in the pulmonary arterioles was assessed. The I score was significantly higher in the AOB_63_ group compared with the C group (2.95±0.91 vs. 0.06±0.04, *P*<0.05). Compared with the AOB_63_ group, the I score in the AOB_63_/ATOR_63_ group was significantly attenuated (0.66±0.40 vs. 2.95±0.91, *P*<0.05, Fig 6).

**Fig 7:**
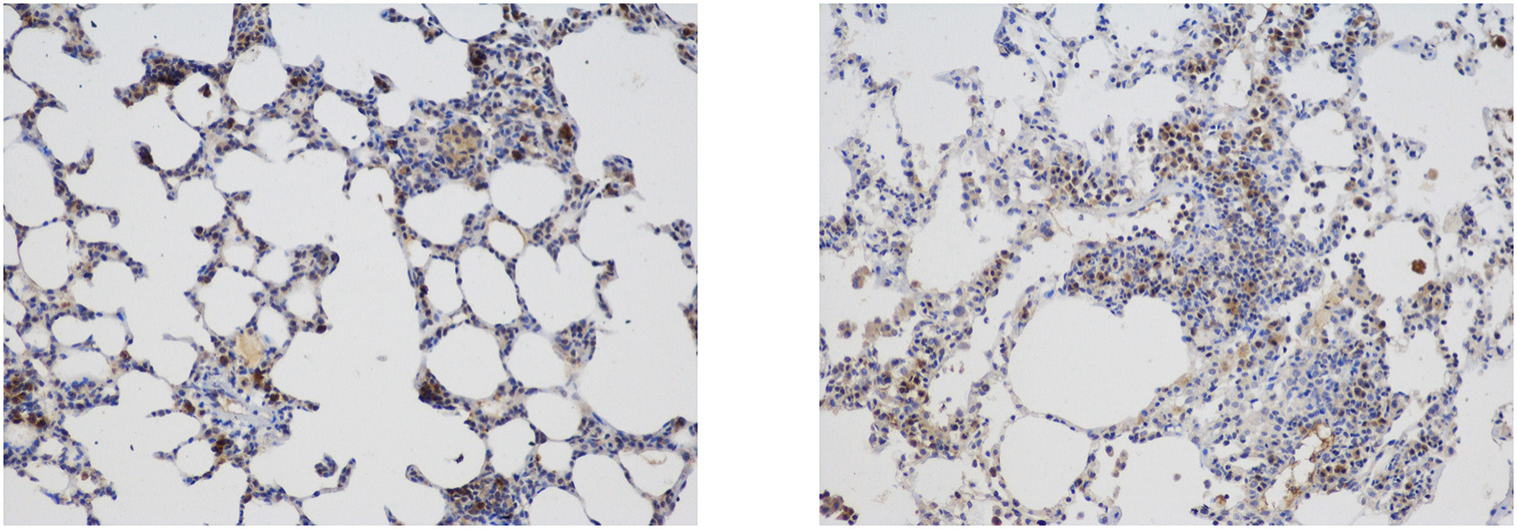
Distribution of Neutrophil Granulocytes Labeled with LY-6G. Immunohistochemical staining (magnification 200×) revealed that the inflammatory cells were distributed in the pulmonary alveoli, the vessel lumen, the vessel wall and the pulmonary interstitium.

**Fig 8:**
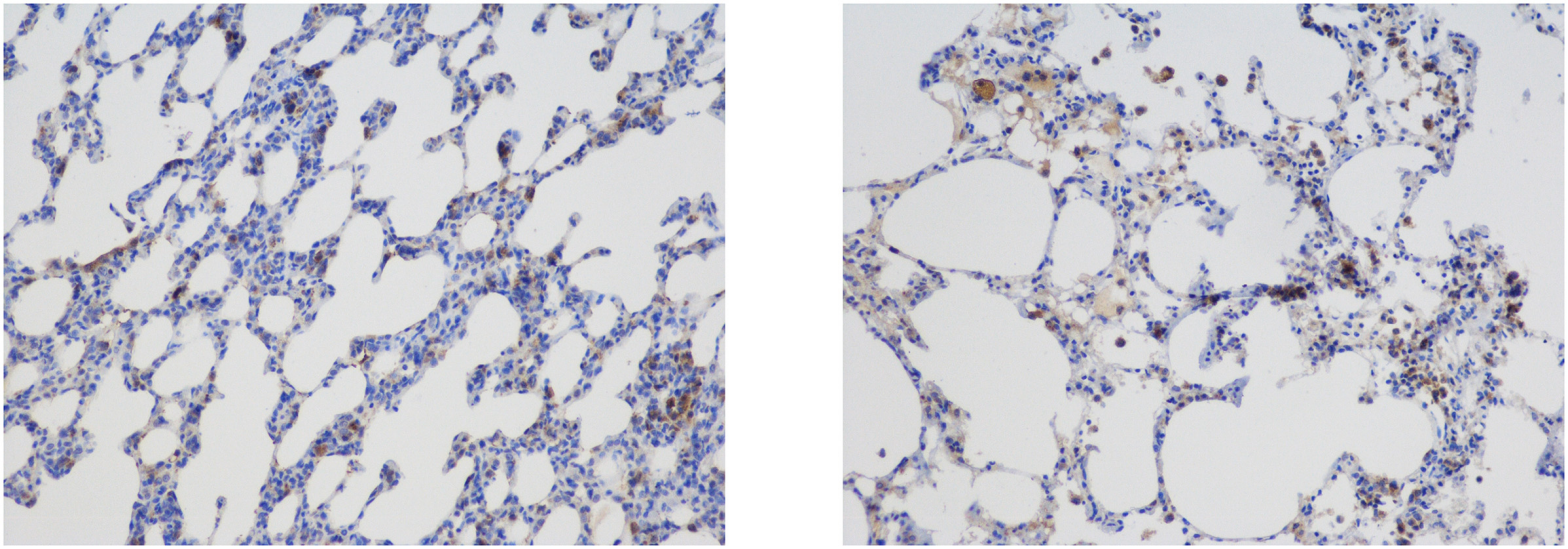
Distribution of Macrophages Labeled with Mac-2. Immunohistochemical staining (magnification 200×) revealed that the inflammatory cells were distributed in the pulmonary alveoli, the vessel lumen, the vessel wall and the pulmonary interstitium.

The inflammatory cells were distributed in the pulmonary alveoli, the vessel lumens, the vessel wall and the pulmonary interstitium (Figs 7 and 8).

### Atorvastatin down-regulated the expression of RhoA and Rho-kinase II (ROCK II)

**Fig 9:**
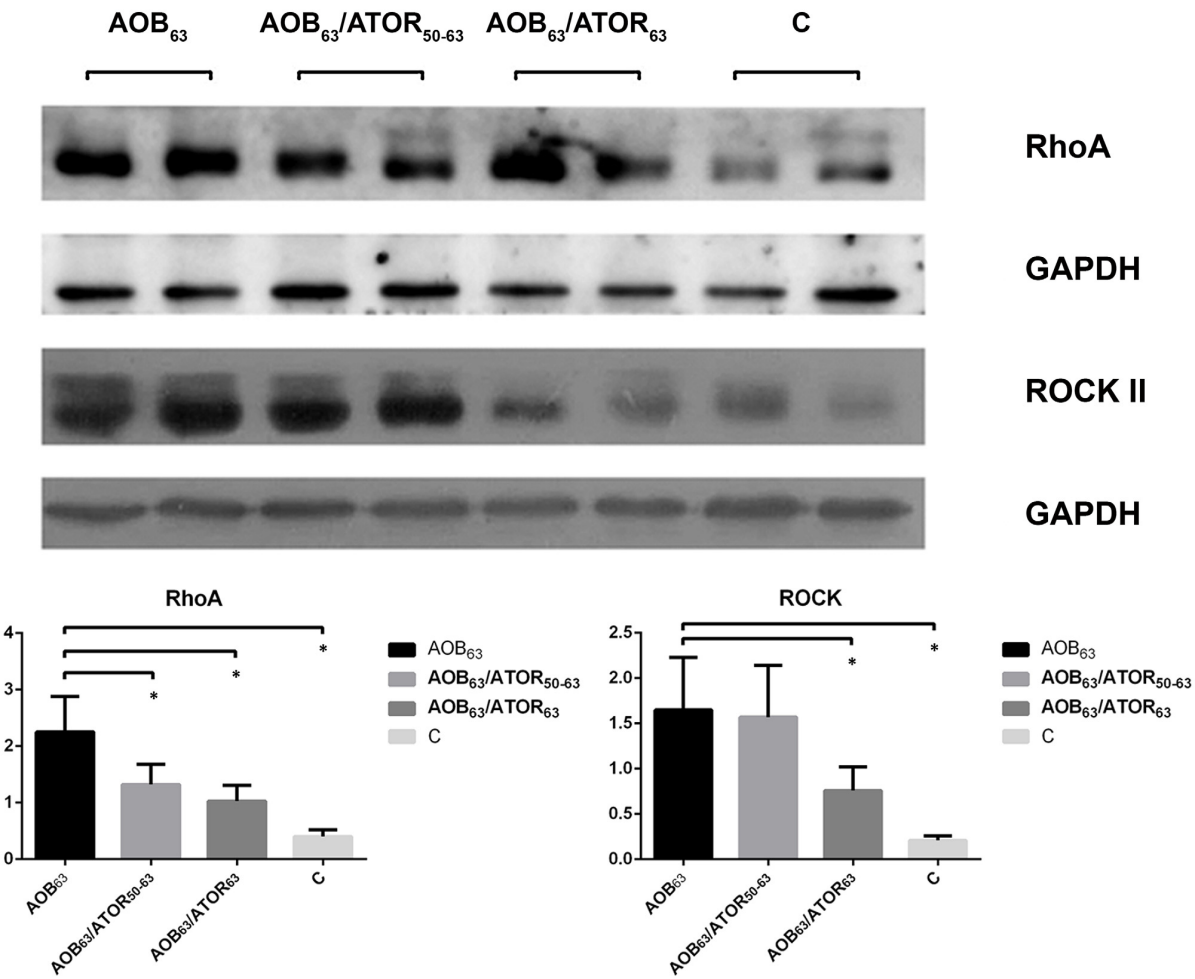
Comparison of Rho A and ROCK II Expression in Lung Tissue among the Groups. A western blot analysis revealed that treatment with atorvastatin resulted in significant decreases in the levels of both RhoA and ROCK II in the AOB_63_/ATOR_63_ group, but a decrease in only RhoA was detected in the AOB_63_/ATOR_50-63_ group. *P<0.05 compared with the AOB_63_ group.

Compared with the control group, the expression of RhoA and ROCK II was significantly increased in the lung of the AOB_63_ group (*P*<0.05). Treatment with atorvastatin resulted in significant decreases in the levels of RhoA and ROCK II expression in the AOB_63_/ATOR_63_ group (both *P*<0.05), but only a decreasing trend in ROCK II was observed in the AOB_63_/ATOR_50-63_ group (Fig 9).

## Discussion

Our results demonstrated pulmonary vascular remodeling in our model of PH induced by aortic banding. Treatment with atorvastatin for 9 weeks resulted in attenuation of PH, pulmonary vascular remodeling and right ventricular hypertrophy. Mechanistically, atorvastatin therapy significantly decreased the expression of RhoA and ROCK II and inflammatory infiltration in the lung. These data provide evidence of the beneficial effects of atorvastatin on pulmonary vascular remodeling in PH induced by aortic banding.

Severe PH and significant increases in the RV and LV weights were observed in the rats 9 weeks after aortic constriction. Moderate lung edema was present at this time, as indicated by a significant increase in the wet-to-dry lung weight ratio. These results were consistent with those described in a previous report [12]. However, Dai et al [9] observed the establishment of pulmonary hypertension secondary to heart failure 4 weeks after the ascending aorta had been banded using 19-gauge banding. In contrast, in the present study, a 9-week period was required for the development of PH. This difference may be attributed to the difference in the residual open diameter of the aorta.

Pulmonary vascular remodeling is a common finding in severe left heart failure [9,12]. Previous studies reported that the thickness of the pulmonary arteriolar wall was increased [9,12]. In the present study, pulmonary vascular remodeling was evident in small arteries. An imbalance between proliferation and apoptosis of the pulmonary arterial smooth muscle cells and inflammatory infiltration were observed in the PH-LHD model. These findings are consistent with previous observations that proliferation of the lung myofibroblasts contributes to the development of pulmonary hypertension and that inflammatory infiltration may be involved in the vascular remodeling of the lungs of hypercholesterolemic rabbits [11,13]. Treatment with atorvastatin (10 mg/kg/d for 9 weeks) attenuated the increasing wall thickness of pulmonary arterioles and the muscularization of pulmonary arterioles by inhibiting RhoA/Rho kinase. This finding is consistent with the well-documented antiproliferative effect of atorvastatin, which has been attributed to mechanisms that include the inhibition of RhoA/Rho kinase activation [14]. It has been reported that vascular barrier function is reduced in the lungs of rats with left heart failure and that this adaptive response partially compensates for the lung edema. As a result, attenuating vascular remodeling may simply aggravate barrier dysfunction and worsen heart failure [12]. However, we demonstrated that atorvastatin attenuated both left ventricular hypertrophy and lung edema. It is therefore possible that some of the beneficial effects on lung function observed in this study were partially due to an improvement in the left ventricular structure, as indicated by a previous study [15]. Another reason this finding may also be concluded may be that because atorvastatin improves lung vascular barrier function and therefore attenuates lung edema. Only a trend of improved pulmonary vascular remodeling was detected with the reversal protocol. Delayed treatment and inadequate length of therapy may be responsible for the results.

One limitation of the present study is that echocardiography was not conducted. However, a previous study demonstrated that the model used in the present study reflects the characteristics of diastolic heart failure. Molecular differences between different HMG-CoA inhibitors have been reported to contribute to distinct pharmacologic and pleiotropic effects [16]. Thus, another limitation of the present study is that we did not compare the effectiveness of hydrophilic and lipophilic statins for the attenuation of pulmonary vascular remodeling.

In conclusion, the present study demonstrates that atorvastatin can prevent pulmonary vascular remodeling in a rat model of pulmonary hypertension induced by aortic banding by down-regulating the expression of RhoA/Rho kinase, inhibiting the proliferation and increasing the apoptosis of pulmonary arterial smooth muscle cells and attenuating the inflammation of pulmonary arteries.
